# Late presentation of arrhythmogenic right ventricular cardiomyopathy: a case report

**DOI:** 10.4076/1752-1947-3-7235

**Published:** 2009-08-04

**Authors:** Georgios I Papaioannou, Theodoros Apostolopoulos, Sotiria Stambola, Antonios Zilidis, John Gialafos

**Affiliations:** 1 Cardiac Catheterization Laboratory, Athens Medical Center, Athens, Greece; 2 Department of Cardiology, Division of Electrophysiology, Athens Medical Center, Athens, Greece

## Abstract

**Introduction:**

Arrhythmogenic right ventricular cardiomyopathy is an inherited myocardial disease affecting predominantly young people and manifests as sustained ventricular tachycardia with left bundle branch block morphology, sudden death or isolated right or biventricular heart failure. However, its first manifestation as sustained ventricular tachycardia in older patients without preceding symptoms of heart failure is infrequent. To our knowledge, our patient is among the oldest reported in the literature presenting with ventricular tachycardia because of arrhythmogenic right ventricular cardiomyopathy without preceding symptoms of heart failure.

**Case presentation:**

We present an unusual case of a very late presentation of a right ventricular cardiomyopathy in a 72-year-old white Caucasian man. The patient was admitted with symptoms of weakness, dizziness and chest discomfort for several hours. His electrocardiogram showed a wide-complex tachycardia with left bundle branch block morphology and left axis deviation. Because of continuing hemodynamic instability, the patient was cardioverted to sinus rhythm with a single 300 J shock. His post-cardioversion electrocardiogram, cardiac echocardiogram, coronary angiogram, magnetic resonance imaging and electrophysiological study confirmed the diagnosis of arrhythmogenic right ventricular cardiomyopathy. The patient was treated with an implantable cardioverter defibrillator and discharged on sotalol.

**Conclusion:**

This case report demonstrates that arrhythmogenic right ventricular cardiomyopathy may have a very late presentation and this diagnosis should be considered as a potential cause of sustained ventricular tachycardia of right ventricular origin among the elderly and should be treated accordingly.

## Introduction

Arrhythmogenic right ventricular cardiomyopathy (ARVC) is an inherited myocardial disease affecting predominantly young people. It manifests as sustained ventricular tachycardia (VT) with left bundle branch block (LBBB) morphology, sudden death or isolated right or biventricular heart failure with the majority of cases been diagnosed before the age of 40, while heart failure symptoms and signs typically appear later in life. Its first presentation as sustained VT in older patients without preceding symptoms of heart failure is infrequent.

## Case presentation

A 72-year-old white Caucasian man, without prior history of heart disease was admitted with symptoms of weakness, dizziness and chest discomfort for several hours. Physical examination revealed low blood pressure (85/50 mmHg) and a weak, regular and rapid pulse. The electrocardiogram (ECG) showed a wide-complex tachycardia with LBBB morphology and left axis deviation. Because of continuing hemodynamic instability, the patient was cardioverted to sinus rhythm with a single 300 J shock.

**Figure 1:**
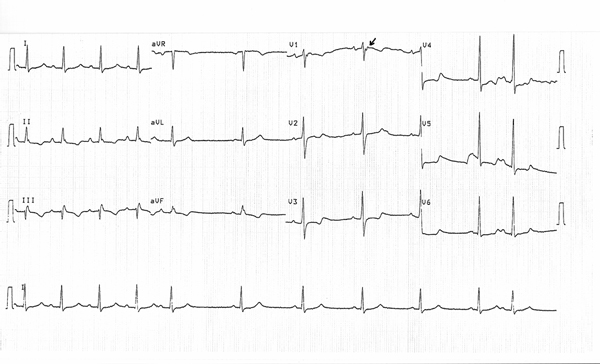
**Patient's electrocardiogram after successful cardioversion**. The QRS duration in leads V_1_ to V_3_ is greater than 110 ms and there is an evident epsilon wave in V_1_ (arrow).

The ECG during the episode of the tachycardia was consistent with sustained VT of right ventricular origin. Post-cardioversion ECG showed negative T-waves at the inferior and precordial leads with a QRS duration of 110 ms and the presence of an epsilon wave in V_1_ (Figure 1).

**Figure 2:**
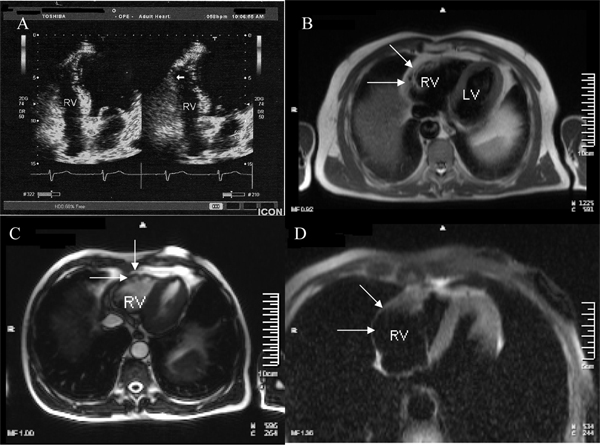
**Panel **(A)** Cardiac echocardiogram (4 chamber view) shows slightly enlarged right ventricle with local dyskinesia and diastolic bulging of the free wall (arrow)**. Panel **(B)** Axial T1 weighted spin echo image shows diffuse hyperintense signal (arrows) in the free wall of the right ventricle. Panel **(C)** T2 weighted cine image with demonstration of multiple small aneurysms at the free wall of the right ventricle (arrows). Panel **(D)** Using an axial T1 weighted spin echo image with fat suppression, the hyperintense signal in the right ventricle wall was suppressed and identified as fatty tissue (arrows).

Chemistries were normal with the exception of troponin I which was positive. The patient was subsequently started on metoprolol. Continuous ECG monitoring revealed multiple episodes of non-sustained VT. Cardiac ultrasound examination showed a normal left ventricle and a slightly enlarged right ventricle with local dyskinesia and diastolic bulging of the free wall (Figure 2A).

As a diagnosis of ARVC was most likely, the patient underwent simultaneous cardiac catheterization and electrophysiological study. His coronary arteries and left ventricle were normal. Right ventriculography in the right anterior oblique view confirmed the local hypokinesia of the free wall with diastolic bulging. During the electrophysiological study, a sustained VT was provoked with identical morphology to the one on admission. The VT responded to antitachycardia pacing and the defibrillation threshold was 17 J.

A magnetic resonance imaging (MRI) scan that followed revealed diffuse areas of fat tissue at the right ventricular wall especially localized at the free and lateral segments, while multiple small aneurysms were also present (Figure 2B-D). Right ventricular ejection fraction was 30%. Left ventricular free wall and intraventricular septum were free of disease. Finally, a signal-averaged ECG was positive for late potentials. Based on the clinical presentation and subsequent work-up, a definite diagnosis of ARVC was made. The patient was treated with an implantable cardioverter defibrillator (ICD) and discharged on sotalol.

## Discussion

ARVC is an inherited myocardial disease primarily affecting the right ventricle and is characterized by the gradual replacement of myocytes by adipose and fibrous tissue. It affects young people and may cause sudden death, especially during athletic activity [1]. Diagnostic criteria for ARVC were proposed in 1996 and include major and minor criteria [2]. Our patient had two major (epsilon wave in lead V1 and MRI findings of multiple localized aneurysms of the right ventricle) and five minor criteria (LBBB type ventricular tachycardia, inverted T waves in precordial leads, regional right ventricular hypokinesia, reduction of right ventricular ejection fraction and late potentials on single-averaged ECG), making the diagnosis of ARVC definite. Despite the extensive T wave changes recorded in the inferior and precordial leads, our patient only had evidence of fibrofatty tissue at the right ventricle, while there was no indication of left ventricular involvement from MRI at the time of presentation.

ARVC manifests as sustained VT with LBBB morphology, sudden death or isolated right or biventricular heart failure. At least 80% of cases are diagnosed before the age of 40, while heart failure symptoms and signs typically appear in the fourth and fifth decades of life. To our knowledge, our patient is among the oldest reported in the literature presenting with VT because of ARVC without symptoms of heart failure.

Although the inheritance pattern is typically autosomal dominant, autosomal recessive ARVC (Naxos disease) with a characteristic phenotype and mutation of plakoglobin, a protein that forms cell-to-cell junctions, is more prevalent in Greece [3]. However, even this recessive form of ARVC almost always manifests before the age of 40. Our patient had never experienced any symptoms that could be related to ARVC and he lacked any specific phenotype or family history that could raise the suspicion of ARVC.

The management of patients with ARVC is targeted toward prevention of sudden cardiac death and treatment of symptoms of heart failure in the case of biventricular involvement. While antiarrhythmic medications including beta-adrenergic blocking agents sotalol and amiodarone can be used to prevent recurrent cardiac arrhythmia, treatment with ICD should be considered in individuals at high risk [4,5]. Radiofrequency ablation [6,7] can be attempted in patients who are unresponsive or intolerant to antiarrhythmic drugs but is frequently unsuccessful and may require multiple attempts because of the patchy nature of the disease. Individuals with ARVC should be prohibited from vigorous exercise and after ARVC is diagnosed, all first-degree relatives should be screened.

## Conclusion

Our case demonstrates that arrhythmogenic right ventricular cardiomyopathy may have a very late presentation and this diagnosis should be considered as a potential cause of sustained ventricular tachycardia of right ventricular origin among the elderly.

## Consent

Written informed consent was obtained from the patient for publication of this case report and any accompanying images. A copy of the written consent is available for review by the Editor-in-Chief of this journal.

## Competing interests

The authors declare that they have no competing interests.

## Authors' contributions

All authors participated in the management of this patient. The first author prepared and revised the manuscript and all authors approved the final draft.
